# Rewiring of the Endocrine Network in Triple-Negative Breast Cancer

**DOI:** 10.3389/fonc.2022.830894

**Published:** 2022-06-30

**Authors:** Kaixuan Li, Dongjiang Zong, Jianrong Sun, Danxiang Chen, Minkai Ma, Liqun Jia

**Affiliations:** ^1^ Department of Integrated Traditional Chinese and Western Medicine Oncology, China-Japan Friendship Hospital, Beijing, China; ^2^ Beijing University of Chinese medicine, Beijing, China; ^3^ Emergency General Hospital, Beijing, China; ^4^ School of Clinical Medicine. Beijing University of Chinese Medicine, Beijing, China; ^5^ Department of Breast Surgery, The First Affiliated Hospital of Wenzhou Medical University, Wenzhou, China; ^6^ Department of Integrated Traditional Chinese and Western Medicine Oncology, The Fourth Central Hospital, Baoding, China

**Keywords:** triple-negative breast cancer, endocrine strategy, steroid hormone, steroid hormone receptor, endocrine responsiveness

## Abstract

The immunohistochemical definition of estrogen/progesterone receptors dictates endocrine feasibility in the treatment course of breast cancer. Characterized by the deficiency of estrogen receptor α, ERα-negative breast cancers are dissociated from any endocrine regimens in the routine clinical setting, triple-negative breast cancer in particular. However, the stereotype was challenged by triple-negative breast cancers’ retained sensitivity and vulnerability to endocrine agents. The interplay of hormone action and the carcinogenic signaling program previously underscored was gradually recognized along with the increasing investigation. In parallel, the overlooked endocrine-responsiveness in ERα-negative breast cancers attracted attention and supplied fresh insight into the therapeutic strategy in an ERα-independent manner. This review elaborates on the genomic and non-genomic steroid hormone actions and endocrine-related signals in triple-negative breast cancers attached to the hormone insensitivity label. We also shed light on the non-canonical mechanism detected in common hormone agents to showcase their pleiotropic effects.

## Introduction

Breast cancers predominantly occur in female patients, and the hormonal milieu lies at the root of the considerable impact on the etiology and pathogenesis. Given the genetic heterogeneity and aberrations in breast cancer, the clinically generalized standard for subtype distinction is based on the expression of estrogen receptor α (ERα), progesterone receptor (PR), and human epidermal growth factor receptor 2 (Her2). The abovementioned molecular biomarkers are also barometers for therapeutic response assessment; ERα indicates a preference for estrogen blockage, and overexpression of Her2 suggests target immunotherapy.

Triple-negative breast cancer, burdened with more than half of the morbidities in about 15-20% of all breast cancer cases, holds the most disappointing survival rates, in which resistance could partly be seen in any targeted therapy in the absence of target receptors (1). The death rates in an epidemiological survey reached up to 40% in the chemotherapy-treated cohorts, and premenopausal female patients with hormones in abundance were the dominantly susceptible population (2). Besides, deficiency of ERα, PR, and Her2 deprived the patients of the benefits of endocrine therapy and limited the response to cytotoxicity chemotherapy. And the assignment of chemotherapy was insufficient to weaken the aggressiveness of the cancer with five-year survival rates less than one-third after adjuvant chemotherapy (3). Thus, the anchorage-dependent treatment concept based empirically upon the expression of target hormone receptors compromised and arrested the promising treatment applications (4).

Typically, endocrine agents are applied to ERα-positive breast cancer comprised of selective ER modulators (SERMs), selective ER degraders (SERDs), and aromatase inhibitors (AI), which are committed to regional recurrence suppression and long-term survival benefit. As evidenced by the fact that ERα-negative breast cancer sheltered from endocrine therapy previously acquired arousable sensitivity to tamoxifen (5), it was implied that the endocrine response promised to be a novel mechanism in TNBC, which the absence of hormone receptors could not overshadow. Thus, endocrine strategies in TNBC were rewired, and the underlying signaling cascades triggered downstream were found to be significant.

The appreciably rising hormone receptors, such as glucocorticoid receptors (GR), androgen receptors (AR), and truncated isoforms of ERs, orchestrate crucial contributions to endocrine response in TNBC, licensing the alternative endocrine strategies to stretch beyond the traditional ERα-blockage orientation. Apart from the familiar mechanism of the estradiol-ER complex transported into the nucleus, rapid hormone effects mediated by membrane receptors have gained increasing attention, which were involved in non-genomic alterations and have the potential to be endocrine-associated targets (6).

We could embark upon recapitulating the hormone-related mechanisms and pathways to optimize the endocrine management of ERα-negative breast cancer, particularly in TNBC.

## Steroid Hormone Actions In TNBC

**Figure 1:**
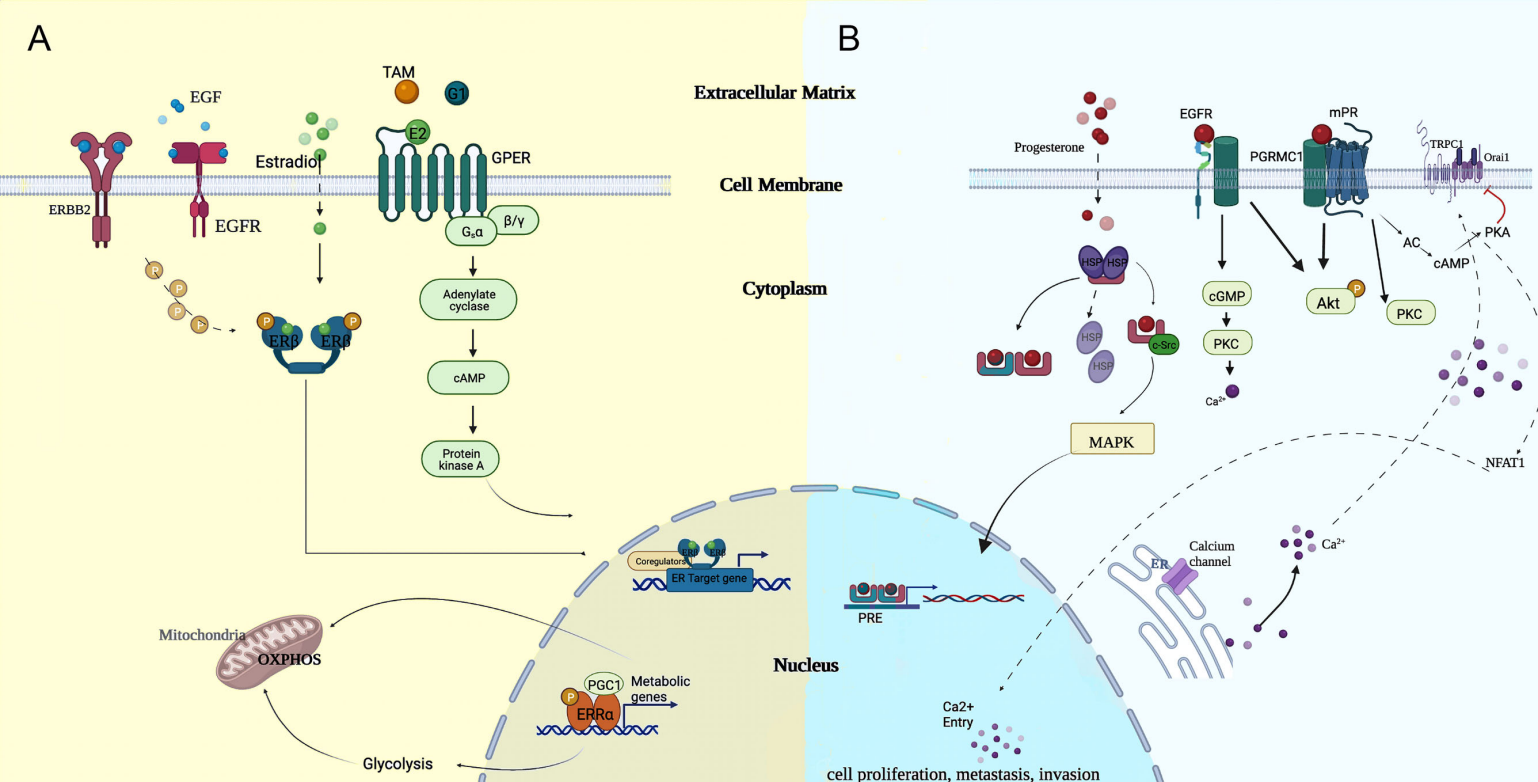
Potential modes of genomic and non-genomic approaches of **(A)** estradiol and **(B)** progesterone, which cooperated in regulating the carcinogenesis process. cGMP, cyclic guanosine monophosphate; EGF, epidermal growth factor; ER, endoplasmic reticulum; MAPK, mitogen-activated protein kinase; NFAT1, nuclear factor of activated T cell; OXPHOS, oxidative phosphorylation; PGC1, proliferator activated receptor-gamma co-activator 1; PKC, protein kinase C; PRE, progesterone reactive element; TAM, tamoxifen.

In general, invasive capacity triggered by steroid hormones was reported to be nuclear receptor-dependent. However, hormone-responsive breast cancers share a common transduction signaling pathway with ERα-negative breast cancers, and the concept in both of them was not mutually exclusive. Manifold resident hormone receptors and recruited circulating hormones enacted their roles in the ERα-independent carcinogenesis process of TNBC. Thus, hormone disequilibrium is pivotally in TNBC as in luminal breast cancers (7). In **Figure 1**, we provide a schematic summary centered around estradiol and progesterone and how they functionally exert transcriptional regulations.

### Estrogenic Activity

Peripheral circulating steroid hormones, specifically estrogens, released from their organs, contributed to the tumorigenic effects in patients with breast cancer. Estrogen was present in three primary forms: estrone (E1), estradiol (E2), and estriol (E3) (8). E2, the major isoform, was biosynthesized by aromatase from the androgenic precursors (9). Lipophilic-natured E2 could mediate either genomic signaling *via* classical nuclear-initiated receptor actions (through ERα and ERβ) or non-genomic signaling *via* membrane surface and intracellular receptors (10). Typically, the combined E2-ER complexes exert estrogen-like effects by binding to estrogen response elements (EREs) at the promoter of targeted genes.

Albeit with the absence of ERα, estrogens and xenoestrogens have been identified to act on the tumor microenvironment *via* their corresponding receptors. E2 functions in the trigger of “don’t eat me” signaling by strengthening CD47-SIRPα interaction and skewing the antiphagocytic effect of the M2 microglia (5, 11). In addition, E2 depletion was of therapeutic value evidenced by the reportedly increasing tumor cell tropomyosin kinase receptor B (TrkB) signal regulated by E2 in premenopausal TNBC patients and thus reduced the risk of brain metastasis (BM) (12, 13). The preclinical model demonstrated that E2-dependent upregulation of brain-derived neurotrophic factor (BDNF) in ERα-positive reactive astrocytes and subsequent activation of TrkB elucidated the carcinogenic role of estrogen in TNBC. Compared to mice with exhausted E2, E2 sheltered TNBC cells from the oncosuppressive effects of the defensive system (13).

Sartorius et al. advanced a novel approach of endogenous E2-mediated brain metastatic colonization, where upregulation of characterized EGFR ligands stimulated by the paracrine effects of E2 in an estrogen-responsive brain microenvironment sensitized the EGFR downstream pathway (14). These studies revealed the E2-fueling function and broadened the therapeutic implications of the tumor immune microenvironment (TIME), such as ER-positive mesenchymal cells and tumor-associated macrophages (TAMs). Since E2 can facilitate the process of immune escape in the BM of TNBC, it could be considered a candidate for macrophage-targeted therapies in primary breast lesions.

The neuroprotective and anti-inflammatory effects of estrogen in CNS were mediated by the rapid activation of ERK1/2 independent from the ER-ERE canonical pathways (15). The tumor microenvironment in CNS evolved and developed an immune-suppressive property during BM (16), and the TNBC subtype was also inclined to the immuno-evasion phenotype compared to luminal subtypes (17). The tumor microenvironment in TNBC remained unknown with the condition to trigger the anti-inflammatory and immunosuppression capacity of E2 just as BM was, which merited research.

### Progesterone

Another principal steroid hormone, progesterone (P4), composed of classical activation of nuclear actions and non-classical activation of membrane actions, applies its progestogenic effect to downstream effector targets similar to estrogen (18, 19). Thus, the progesterone receptor (PR) could be divided into the nuclear progesterone receptor (nPR) and non-genomic receptors, such as membrane progesterone receptors (mPRs) and progesterone receptor membrane component 1 (PGRMC1) (20).

Once evidenced by the whole genomic effect of progesterone when PR reactivated in TNBC, P4 upregulated the expression of genes detrimental to cell proliferation and invasion and consistently dampened the genes in the maintenance of genomic stability (21). Anticancer properties of P4 target genes were identified in endocrine-insensitive TNBC, which supplied a novel option for endocrine strategies. Besides, progesterone metabolites, named 5α-dihydro-progesterone (5αP) and 3αHP, exerted the opposite effects on the evolution of TNBC, which is significantly enhanced by 5αP while suppressed by 3αHP (22), while a high concentration rate of 3αHP:5αP was responsible for the preservation of physiological conditions in a nontumorous environment.

In summary, progesterone circumstantially exerted an antitumor effect in TNBC, and more evidence was required to unravel the discrepancy in the role of the non-genomic membrane progesterone receptor in TNBC.

## Nuclear Hormone Receptors Acting as Transcription Factors

Although TNBC cells do not express ERα and PR, other hormone transcription factors synergizing to initiate endocrine signaling cascades are equipped with the capacity to activate endocrine network transponders *via* ERα-independent pathways, such as androgen receptors (ARs) (23, 24), glucocorticoid receptors (GRs) (25–27), and distinct isoforms of ER. As listed above, ERα-negative results in immunohistochemical staining (IHC) could not be responsible for void endocrine efficacy in TNBC.

### Estrogen Receptor α-36

As a specific isoform of traditional ER-α (ER-α66), ER-α36 was first mentioned by Wang et al. in 2005 with a truncated length of 36 kDa (28). Compared to the familiar ER-α66, ER-α36 could be expressed concurrently in TNBC and mediate estrogen signaling transduction (29), which was first identified to express a specific transcriptomic signature in TNBC (30). Although both transcriptional activation domains are lacking, the retained DNA-binding domain and dimerization-binding domain endowed ER-α36 with a dominant-negative regulation of the transactivation functions signaled through ER-α66 and ERβ (31). ER-α36 was involved in the genomic mechanism of carcinogenesis and invasion albeit with an inferior proportion than its non-genomic actions. The integrity of the essential domain potentiated ER-α36 to dimerize with ERα and translocate into nuclear ERα to initiate nuclear actions. However, ambiguous conclusions underlie the counteracting force between ERα36 and ERα66. Wang et al. revealed that tamoxifen functioned as an ERα36 agonist to upregulate the expression of aldehyde dehydrogenase 1A1 (ALDH1A1) in CSCs, which plays a pivotal role in the maintenance of cancer stemness properties (32). This finding enriched the genomic mechanism of ERα36 independent of ERα66 status in response to E2 and tamoxifen. It was recognized that truncated ERα36 and ERα66 were mutually interactive and restrictive. A previous study demonstrated the regulatory role of ERα36 to downregulate ERα66 expression *via* upregulation of EGFR (33, 34). Besides, ERα66 could interact with the ERE-half site in intron 1 of the ESR1 coding region and thus lead to the inhibition of ERα36 transcription activity (35). In addition, a recent study demonstrated that ERα36, in collaboration with GPER, inhibited NFκB-mediated pro-inflammatory activity and the expression of downstream TNFα and IL-6 in TNBC (36).

### ERβ

Compared to the indicative role of ERα in endocrine intervention efficacy, ERβ was expressed in cancer stem cells, normal epithelium cells, stromal cells, and even TNBC, the distribution of which was thought to lack specificity (37–39). ERα and β have been identified as homologous, sharing a high degree of similarities in DNA-binding domains despite their respective ligand-binding distinctions and transcriptional activating function domains. All the isoforms of ERβ (ERβ2-5) except for ERβ1 were disabled to combine with bridging ligands autonomously and could only be dimerized with ligand-binding ERα/β1 to activate the negatively estrogen-related signaling pathway. The global genomic landscape regarding the interplay between ERβ and the oncogenic genome in breast cancer revealed that ERα and β intersect extensively with each other in target gene regulation. Because of the spatial proximity to the mitochondrion, ERβ was taken to contribute to mitochondrial DNA-encoded genes’ function through an ERE-like sequence (29).

As evidenced by previous literature, ERβ was envisioned as a bifacial factor predictive of breast cancer survival. E2/ERβ-mediated aggressiveness and stemness properties in TNBC could be in part explained by downstream actions which promote EGFR, VEGF, amphiregulin, and Wnt-10β secretion (22). The adverse effect caused by ERβ could be reversed by tamoxifen in ERα-negative tumors. Besides, the presence of ERβ improved tamoxifen-treated ERα-positive breast cancer patients. As a kind of classical SERM, fulvestrant inhibits growth-stimulating effects of ERβ by negatively regulating DNA methyltransferase (DNMT), widening the scope of the intent-to-treat population to ERα-/ERβ+ breast cancer (40).

When concurrent with ERα, ERβ commonly shows an antagonism against tumor proliferation and invasion (23), reflected in a restrained output of ERα and its mediated transcriptional activities by the decreasing recruitment of c-Fos and c-Jun to the estrogen response promotor (24). *In vivo* experiments substantiated the oncosuppressive role of ERβ by the fact that its loss activated the overexpression of ERα and inducted an aggressive phenotype (32). Mechanism investigation revealed that the downregulation to cell cycle repressive tumor protein 53-induced nuclear protein 1 (TP53INP1) by ERα could be totally reversed by ERβ and thus considerably decreased a few cyclins, such as CCNA2, CCNB1, CCNB2, CCND1, and CCNF as a result (26). As a regulatory genomic analysis manifested, the gene profiles which were promoted by ERα while inhibited by ERβ were mainly concentrated in the function of “cell cycle”, “xenobiotic metabolism”, and “ion transport”, and genes in these pathways primarily were evidenced as tumorigenesis biomarkers.

In addition to the interplay with ERα, the crosstalk with AR underlies the contribution made by ERβ to anti-androgen efficacy improvement. The heterodimer constituted by ERβ competitively binding to AR impeded the development of the AR-AR homodimer and blocked the subsequent PI3K/AKT oncogenic signaling pathway. Song et al. found out that ZEB1, an invasion promotor expressed in concert with E-cadherin, was inhibited by ERβ and thus abrogated its original aggressiveness phenotype (28).

ERβ acted more on the proliferation of cancer stem cells than ERα, given the proportion towards estrogen response. Yet, its impact on TNBC progression is just beginning to be explored. Three of the exclusively regulated genes by ERβ were associated with lipid and cholesterol metabolism. Alexandrova et al. identified the ERβ-induced inhibition of cholesterol biosynthesis mediated by miR-181a-5p in a small non-coding RNA profile towards TNBC, which testified the cholesterol metabolism correlation (41).

Most findings proposed that ERβ-mediated tumor inhibition was through cell arrest at the G1 phase and the downregulated cyclins. Besides, the negative regulation of ERβ towards tumor suppressor genes could be instantiated in the contribution to stability failure of EGFR and suppression towards p53 mutagenesis. The accumulating evidence indicated that Erβ, devoted to diminishing the genes encoding key components, positively related to TNBC aggressiveness. Moreover, the interplay of ERβ and the intranuclear molecular chaperone instigates the process of gene regulation, RNA splicing, and chromatin remodeling at the transcriptional and post-transcriptional level, which was validated by the association between ERβ and the polycomb repressor complexes 1 and 2 (PRC1/2) in the process of cholesterol biosynthesis inhibition in interaction proteomics (42, 43).

The adaptive strategy in the context of a double-faced role of ERβ still hangs in doubt. A phase II clinical trial (NCT03941730) aiming at the conundrum above was conducted in the ERβ-positive TNBC population, which traded off the activation of the tumor-suppression effect by E2 against the refrainment of the tumor-promoting effect by tamoxifen regarding the pros and cons of ERβ.

### Glucocorticoid Receptors

As one of the nuclear hormone receptors, GRs act as ligand-activated transcription factors and require little agonist dependency on the ligand for signaling activities (26). Ligand-activated GR phosphorylation modified feedforward signaling loops with sensory input of TME-derived stress signals for the persistent activation of stress signaling pathways to actuate advanced cancer biology in TNBC. Besides, a convergence of host and tumorous stress stimuli could activate a p38MAPK-led ligand-independent phospho-GR and form a signal amplifier mediated by the positive feedforward control towards p38MAPK (44). Nuclear translocation upon activation, followed by the combination of glucocorticoid response elements (GREs) and other transcription factors like NF-κB and AP1, was linked to regulation of functional gene expression and risk of developing aggressive modalities of breast cancer.

The GR functioned as a tightly regulated homeostatic machinery of tumor microenvironment homeostasis disruption, reflecting on the stress signaling factors and pathways triggered by solid tumor necrosis and tissue remodeling, which was indicative of the fact that any modifications in the alteration of GR activity would cripple the feedback regulation and contribute to pathogenesis (45).

Overexpression of GR in untreated TNBC predicted a poor prognosis (HR=1.73), and 24 pS134-GR-dependent genes were linked to inhibition of apoptosis in breast epithelial cells (46). However, the presence of GR provided a feasible target towards a series of advanced tumor phenotypes, chemoresistance, and anti-androgen resistance in the treatment of TNBC (47, 48).

### Androgen Receptor

**Figure 2:**
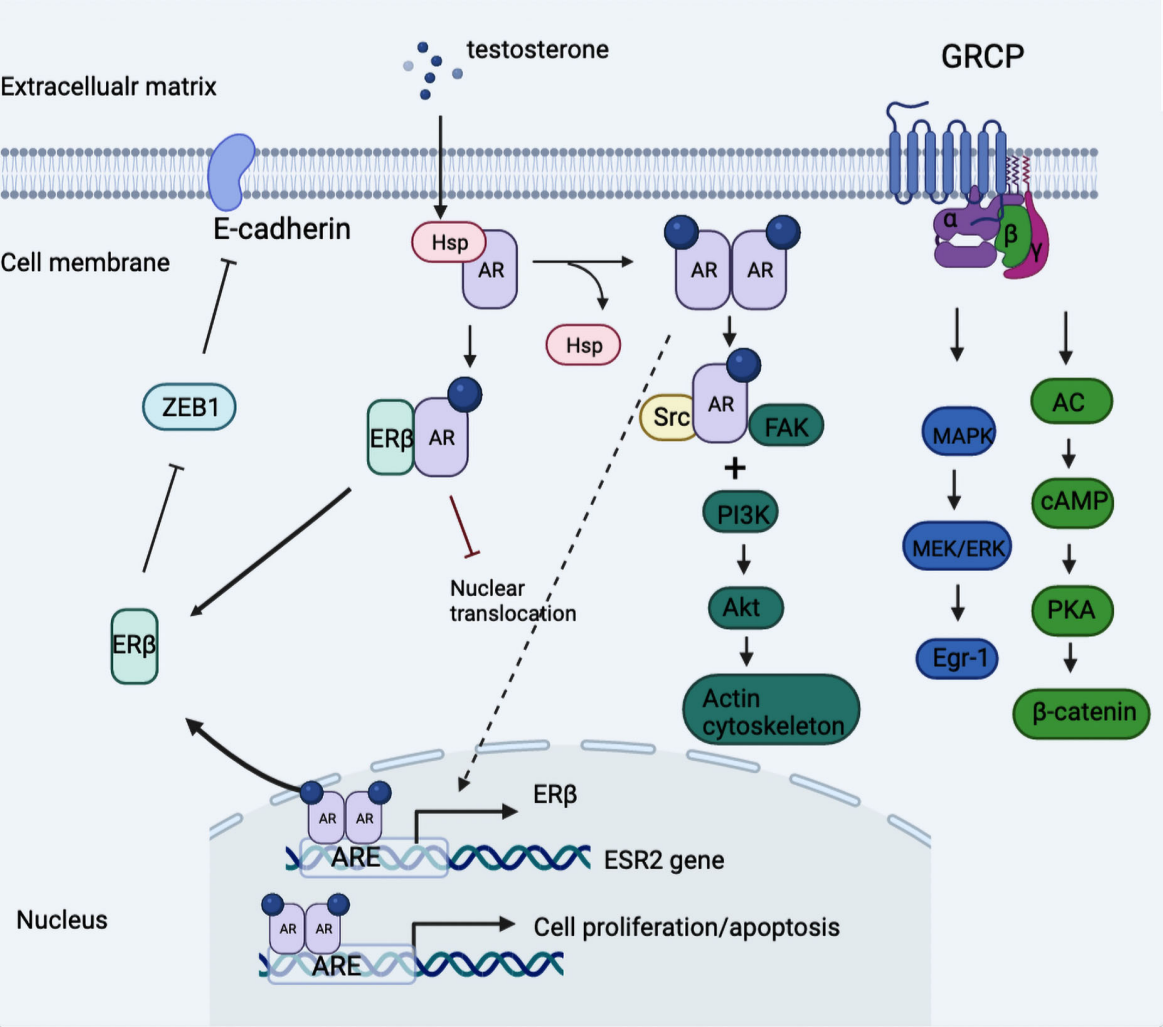
Model of androgen-induced genomic and non-genomic actions in TNBC cells. Classical AR was divorced from the HSP and formed the homodimer once activated by a ligand, which was then transferred into nuclear actions and binding to the promotor of ESR2 gene linked to regulation of ERβ expression. Further, the expressed ERβ dimerized with AR then impeded the nuclear translocation of AR-AR homodimer and thus blocked downstream oncogenic signaling. In addition, androgen stimulation activated the classical AR/Src complex assembly, rapidly recruiting PI3K and FAK, which triggered the downstream phosphorylation and the consequent cytoskeleton changes. Besides, the G protein-coupled form of AR activated downstream MAPK/MEK/ERK signaling and induced the phosphorylation of cAMP and PKA. ARE, androgen receptor element; Egr-1, early growth response 1; GRCP, G protein coupled receptor; HSP, heat shock protein; ZEB1, zinc finger E-box binding homeobox 1.

The presence of an androgen receptor subdivided TNBC into quadruple-negative breast cancer and AR-positive TNBC, the latter of which harnessed a different dependency on the androgen receptor, which was more ubiquitously expressed than ER and PR (49, 50). In **Figure 2**, we summarized the androgen-induced genomic and non-genomic actions in TNBC cells.

Estrogen response activity *via* estrogen-regulated genes was mainly determined by the conditionally essential FOXA1 in the form of silencing modifications that disrupt all the ERα-related chromatin and transcriptome activities (51). Albeit with the role of FOXA1 as a pioneer of ERα actions and the high-baseline estrogen milieu in female patients, the expression of FOXA1 in ERα-deficient BCs attained approximately 30% (52). The fact that more than 80% of FOXA1-attended carcinogenic events did not overlap with ERα-induced carcinogenesis, increasingly shifted the focus to the additional endocrine mechanism beyond its traditional Erα dependency. Robinson et al. reported the participation of FOXA1 in the transactivation of an AR-mediated downstream program in the molecular apocrine TNBC, which was gathering endocrine-responsive genes resembling the luminal signature of ERα-positive BC (53). FOXA1 rechanneled AR binding sites to the objective domain where ER originally functioned. And thus, AR cistron stood in the way of carcinogenesis by ERα (54) while emulating the ERα-leading carcinogenic program when silencing ERα (55, 56). Although FOXA1 is indeterminate in its role as an endocrine target spot, a battery of findings indicated its prospect to intercept hormone signaling in the prerequisite of negative hormone receptors.

An AR in TNBC cells sufficed to modulate ER-mediated downstream signaling independent of ER, such as the MAPK/ERK and PI3K/Akt/mTOR pathways (57). The intersection between AR- and ER-mediated signal cascades occurred in the AR-binding motif, PTEN, which suffered from upward control by ER, and thus ascribed the antitumor function of AR to the declining output of the PI3K/AKT pathway by upregulated PTEN (58). Conversely, the activating PIK3CA mutations and increase in pAKT were abrogated by AR inhibition, which gave an exposition of the tumor-promoting action of AR. The other mechanism analysis found that the anchoring of AR at an ARE in the promoter of the ERβ gene resulted in the overexpression of ERβ. Besides, synergistic inhibition was observed *in vitro* with combined CDK4/6 inhibition and anti-androgens in luminal and TNBC cell lines (59).

## Non-Genomic Actions

Although with empirically proven predilection of nuclear transcriptional ERα for genomic carcinogenesis, alternate isoforms of canonical receptor induce rapid non-genomic actions (60). Non-genomic actions were inclined to drive a rapid alteration *via* membrane fluidity and the accompanying activation of second messenger pathways, thus mediating various biological responses. For example, the following receptors received increasing attention in the area of investigation.

### ERα36

As an extranuclear-acting isoform of ERα, ERα36 mainly mediates rapid non-genomic actions by converging on two principal signaling pathways, A) direct phosphorylation of the MAPK/ERK pathway by activation of c-Src (61, 62), and B) activation/phosphorylation of the PI3 kinase (PI3K)/Akt axis and inhibition/phosphorylation of glycogen synthase kinase 3β (GSK3β), which secures the stability and nuclear translocation of transcription factor nuclear factor-E2-related factor 2 (Nrf2) (63). Nrf2 enacted its essential role in metabolic reprogramming and antioxidation regulation *via* binding with the antioxidant responsive element (ARE) and subsequently regulating metabolic-associated genes (64). In addition, Zhang et al. demonstrated the mitogen-activated estrogen- and antiestrogen-dependent signaling pathway through the phosphorylation of EGFR and Src by physical interaction (35), which could be suppressed in ER-α36-knocked out TNBC cells using short hairpin RNA with the bypass activation of the PI3K/AKT signaling pathway (65). Besides, the signals which ER-α36 transmitted through the EGFR/HER-2/ERK pathway converged towards cisplatin resistance (66).

### Estrogen-Related Receptor α

The orphan receptor, known as estrogen-related receptor α (ERRα), was highly homologous to classical ERα in the aspect of target nodes, regulatory elements, and sites of action (67). In hormone receptor-negative SKBR3 cell lines, a physiological dose of E2 could motivate the expression of ERRα to regulate estrogen. Together with peroxisome proliferator-activated receptor γ co-activator 1α (PGC-1α), ERRα acted to regulate substantial metabolic-associated molecules. The PGC-1α/ERRα axis has been recognized as the crucial regulator for mitochondrial biosynthesis and function and is pertinent to the Warburg effect and high-energy metabolism (68). The cholesterol-ERRα axis functioned in disturbing purine anabolism and folate metabolism by one-carbon resource suppression. As the endogenous ligand of ERRα, cholesterol initiated an auto-induction loop of ERRα and strengthening of target genes expression *via* the interplay with its co-stimulator PGC-1α (69–71). Furthermore, metabolic reprogramming in an ERRα-dependent manner encompassed increased oxidative phosphorylation (OXPHOS), TCA cycle intermediate levels, and the pentose phosphate pathway, which constituted metabolic vulnerabilities in TNBC (72). ERRα augmented the NADPH level by the process of malate-aspartate shuttle and glucose-6-phosphate dehydrogenase (G6PD), which was further employed to extend the actions of biomass synthesis and ROS detoxification, and orchestrated the malignant phenotypes in TNBC (73–75). Notably, inhibition actions to ERRα, known as the metabolic energy sensor, acted to set back epithelial-mesenchymal transition by directly targeting fibronectin (76).

### G-Protein Coupled Estrogen Receptor

G-protein coupled estrogen receptors (GPER), well established as membrane-bound and cytoplasm-located sex steroid hormone receptors, are yet fully defined in TNBC (77, 78). Particularly remarkable is that SERDs and SERMs with ER-degrading effects were reported to be the agonists of GPER, except for the original E2 ligand, which elaborated the partial mechanism for tamoxifen escape although this effect is not universally accepted. In addition, GPER intermediated the regulation of E2-binding ERRα, and a positive impact to ligand-activated ERRα function was observed in the context of overexpressed GPER, which could be nullified by siGPER by the transfecting plasmid. GPER participated in the ligand-initiating rapid non-genomic actions in TNBC *via* interfacing with phospo-ERK (pERK), phospo-focal adhesion kinase (pFAK), and cell cycle proteins, such as cyclin A and cyclin D1, which retains responsiveness to mitogenic estrogen signaling in the circumstance of hormone repletion (79). A large proportion of analysis revealed that GPER was burdened with maintaining stem cell-like and self-perpetuated properties *via* induced phosphorylation of PKA and BAD-Ser118 in tumor tissues (80) and compromised prognosis and survival in TNBC. The *in vitro* experiment demonstrated that targeting GPER in SKBR3 cell lines endogenously expressing GPER kept tumor cells arrested in the G2/M cell cycle (81), wherein dormant tumors were susceptible to a cytotoxicity effect. Consequently, endocrine therapy is expected to be orientated in the multi-direction blockade on cytoplasmic and intranuclear estrogen receptors.

Based on the extensive studies of GPER-binding endocrine actions, the evidence to date evoked substantially different standpoints which were dissociated from the fixed role as a carcinogenesis promotor and unfolded the antitumor activity of GPER in a diverse collection of tumor responses. To begin with, the mechanism against tumor progression of GPER partly consisted in the attenuation of mitogenesis activity by estrogen and the GPER-mediated stimulation of histone H3 and caspase-3 that subsequently brought about the consequence of cell apoptosis. A recent study elucidated that activation of GPER *via* its specific agonist G-1 suppressed the proangiogenic factor, such as interleukin 6 (IL-6) and vascular endothelial growth factor (VEGF), and NF-kB, and thus directed at the angiogenesis and invasiveness was perceived as the critical conundrum of aggressive TNBC (82).

Discrepancies of GPER-mediated proliferation effects on breast cancers included the growth inhibition of endocrine-sensitive MCF7 cells and the growth-promoting effect of ER-negative SKBR3 cells (83). Notas et al. identified the interplay of ER and GPER in extra-nuclear ER actions *via* the pharmacological approach towards human breast cancer cell lines T47D and MDA-MB-231 (84), which was in concert with the previously reported clinicopathological evidence that correlated ER and GPER expression in breast cancer either in a positive (85, 86) or a negative way (87, 88). In a word, early membrane-initiated actions of estrogens in breast cancer are governed in a complicated manner, in which the effects of GPER on transcription mainly depend on the concurrent activation of ER variants.

### Non-Genomic PR Signaling

For the past several decades, a limited pool of PR approximating to the plasma membrane has been extensively observed to exert rapid progestogenic effects in a canonical PR-independent manner. The non-genomic mediator was predominantly affiliated with the progestin and adiponectin Q receptor family (PAQR), which was constituted by the non-canonical G-protein coupled mPRs (89). The mainstream pathways initiated in rapid non-genomic PR signaling were the PI3K/Akt and Src/MAPK pathways dependent on the direct interaction, which functioned in the progestin-mediated angiogenic switch *via* VEGF secretion and the formation of the advanced metastasis phenotype (90–94). In the absence of classical PR in TNBC, P4 initiated a non-classical membrane signal with the P4-PGRMC1 conjugate in the disservice of intracellular calcium homeostasis in TNBC cell lines, where the P4-dependent Ca2+ mobilization pathway encountered the block of PGRMC1-mediated nuclear factor of activated T-cells 1 (NFAT1) intranuclear downregulation (95). Overexpression of PGMRC1 was further linked with the invasion phenotype and poor prognosis in widened signaling of the PI3K/AKT/mTOR and EGFR pathways (96, 97). Besides, PR could indirectly upregulate the proliferation-associated genes with the STAT3 binding region in their promotor instead of PRE *via* the PR-initiated signaling complex involving Src, ErbB-2, JAK1, and JAK2 (98).

Narayanan et al. indicated that the cytoplasmic pool-localized PRs gathering predominantly in the G1 phase experienced relocalization into the nucleus in the S phase, which partly explained why the non-genomic activity of mPR tightly regulated the transition of the G1/S phase and the subsequent activation of cell cycle proliferation (99).

Research revealed the biphasic effects of progestin and the non-canonical PR complex in the TNBC cell lines. Progesterone suppressed the tumor proliferation and brain metastasis convergently *via* mPR (16) and reversed the mesenchymal to epithelium-like phenotype in the MDA-MB231 cell line. In addition, PGRMC1 was certified to increase the chemotherapeutic resistance and abrogate the apoptosis effect of doxorubicin (100).

### Non-Genomic AR Signaling

AR was generally proposed as an androgen-activated transcriptional factor equipped with genomic and non-genomic actions, the latter of which was mainly specified in this chapter.

G-protein coupled receptors (GPCRs) known to mediate androgen actions *via* the second messenger effect, such as Ca2+ efflux and ERK phosphorylation, were perceived as membrane ARs (mARs).

The evidence so far suggested that androgen-induced mAR responses were circumstantially dependent on different cell subtypes and cellular environments (101–104). Giovannelli et al. identified a classical AR/Src/PI3K complex assembly, which triggered cytoskeleton changes and resulted in motility and migration in TNBC-derived MDA-MB453 cells with different levels of AR expressions (101). In comparison, previous research reported a pro-apoptosis role of membrane-initiated AR in both luminal and TNBC cells contrary to the abovementioned carcinogenesis promotor (102, 105). In detail, the upregulation of intrinsic apoptosis molecules, like Bax, cytochrome C, and caspase 3, were exerted by activation of G proteins and the subsequent MAPK/ERK pathway as well as increases in intracellular zinc concentrations in MDA-MB468 cells (105). Besides, the androgen-stimulated pro-apoptosis mechanism governed by mAR could be reflected in the actin restructuring and stasis *via* AR/Src/FAK/PI3K signaling and downregulation of FAK and Akt (102). The abovementioned androgen-specific zinc transport and pro-apoptotic function of zinc transporter member 9 (ZIP9) was mediated by concentration-dependent zinc transport activity through the stimulatory Gαs protein. In addition, Kalyvianaki et al. comprehensively discussed the alternative forms of mAR through affinity binding with testosterone-BSA, such as G protein coupled oxo-eicosanoid receptor 1 (OXER1) and G protein coupled receptor family C group 6 member A (GPRC6A). They proposed the significance of incorporating these receptors into the design of future therapeutic targets (106). OXER acting as a specific mAR in breast cancer was antagonized by testosterone for its original cytoskeletal re-arrangement, thus modulating the adhesive and migratory capacity (107). GPRC6A, well known as the regulatory element of complex endocrine and metabolic networks, was reported to activate downstream ERK and dampen the output of Egr-1 pathways when binding with Gαi protein (108).

The fact that mAR was trapped in the controversy with divergent evidence suggesting both inhibitory and promotive actions in breast carcinogenesis could be in part restricted to the unintelligible preference of the mAR ligands towards the trigger of non-genomic or genomic actions in breast cancer cells.

### Non-Genomic GR Signaling

GR was well defined in the rapid non-genomic pathway associated with auto-immune diseases and cancers (109). However, little evidence was proposed regarding the non-genomic effects in cancers, especially breast cancers.

Clarisse et al. lately overviewed the non-genomic mechanism of GR-mediated apoptosis in lymphoid malignancies, primarily associated with the consequences of cytosolic K+ and Ca2+ mobilization, and production of reactive oxygen species (ROS) paralleled by the oxidative stress (110). Leis et al. show that GR could counteract the carcinogenic actions of the PI3K/Akt pathway in skin tumorigenesis, and the co-expression of GR and AKT in keratinocytes repressed the AKT-driven tumor pathways (111). They attributed the non-genomic GR-mediated PI3K/AKT downregulation to the transcription-independent activity using the transcriptionally defective GR mutant. The unique mechanism of GR revealed that GRs were dissociated from a complex containing Src upon the combination with GCs, and the release of Src subsequently caused the phosphorylation of iNOS and the activation of the oxidative pathway in a non-genomic manner (112).

## Estrogen Responsive Genes and Signaling Pathways

ER served as a determinant to regulate cell fate, and the downstream cyclin-dependent kinases (CDKs) were expected to participate in the target chain of endocrine blockage. The CDK4/6 antagonists were certified to abrogate the progression of mitogenic activities in TNBC in *in vitro* and *in vivo* studies, which elucidated the nullification of the cell division cycle as the endocrine blockage directed at CDK4/6 was in an ER-independent manner (113).

A distinct TNBC subtype, identified as luminal-AR (LAR), manifested itself in tight relevancy on highly activated hormone-associated signaling pathways, including steroidogenesis, porphyrin metabolism, and androgen metabolism in particular. Comprehensive genomic analysis elaborated the molecular evidence of activated estrogen downstream signaling. It indicated the responsiveness of the LAR subtype towards traditional anti-estrogen/androgen strategies independent of ER status (114), which could be in part explained by the inconsistency between the weakly expressed ER protein and ER-coded gene (115). Besides, Williams et al. identified a 32-gene centroid signature derived from ESR1 (encoding ERα) and its downstream targets gene, thus correlating TNBC with ER response (116). The breast cancer subtype phenotypically recognized as ER-negative was equipped with hormonally transcriptional genes in ER-positive cancers, which could be directed by endocrine strategy in an ER-independent but AR-dependent manner.

## Epigenetic Regulation

In parallel with the deletion alterations of hormone receptors in metastasis during distant dissemination, the regain of hormone receptors *via* modifications in epigenetic regulation should also be acknowledged. Preclinical and clinical studies exhibited a disproportionately higher rate of genetic aberrations in TP53, BRCA1, and EZH2 (117). Yomtoubian et al. found that EZH2 inhibition differentiates EZH2-high basal cells to an endocrine-sensitive subtype by derepressing GATA3, which provided a novel target resensitized to the endocrine agents and unconfined by sole chemotherapy (118). A recent study investigated the inducible regain of functional ERα and AR in the SKBR3 cell line by combining the DNA methyltransferase inhibitor (DNMTi) and histone deacetylase inhibitor, which revealed the availability of endocrine sensitivity in prospect (119).

## Non-Canonical Mechanism of Endocrine Agents

Since the predominant perspectives proposed that the presence of classical ERα and PR was fundamental to endocrine deprivation therapy, previously little evidence linked existing endocrine agents, as well as the hormone signaling transfer system, to the potent research directions and therapeutic target in the triple-negative aggressive subtype of breast cancer, which merited concentration. Steroidal AIs, such as formestane and exemestane, could conquer the endocrine resistance of nonsteroidal AI in an ER-independent but AR-dependent manner for their direct absorption into TNBC, which could be dictated by their androgenic metabolites hindering the accessibility of CCND1 by histone modification in G1/S transition (120).

Gonadotropin-releasing hormone receptors (GnRHR) are ubiquitously expressed in both normal glandular tissue and malignancies. Researchers found that GnRHRs frequently emerged in more than half of TNBC, which were inspired as an immediate target assaulted by GnRH analogs (GnRHa), such as goserelin (121). And a multitude of studies have shown that GnRHa was involved in the disruption of an autocrine stimulatory loop wherein the agonists imposed restrictions on the output of the gonad axis and sheltered the ovarian function from chemotherapy toxicity in ER-negative patients (122–124). In addition to preventing premature ovarian failure for young breast cancer patients, concurrent usage of GnRHa with chemotherapy-induced early-onset suppression of progesterone, and in turn, the level of RANK/RANKL, rallied the sensitivity to chemotherapy attack in TNBC (125). Furthermore, Nishiwaki et al. recently unveiled that raloxifene and bazedoxifene but not tamoxifen acted as ERβ agonists in hepatocellular carcinoma cells to attenuate the transforming growth factorα (TGFα)-induced migration by specifically inhibiting phosphorylation of AKT (126).

Tamoxifen was usually taken as a frequently used anti-estrogen agent, and Morad et al. exploited its potentialities to promote lysosomal membrane permeability irrespective of estrogen receptor status and effectively modulated ceramide metabolism to maximize the cytotoxicity effect (127).

The tamoxifen-regulated transcriptional analysis shows that tamoxifen positively induces pluripotency of breast cancers (128). Further, the clinical ATLAS trial proved that a prolonged tamoxifen regimen achieved clinical benefits *via* an alleged sleeping strategy that put all the cancer cells in the dormant state shunning local or metastatic relapse (129).

However, the controversial mechanism of tamoxifen, whether it acted as an agonist on non-classical ER (such as GPER) or an antagonist on classical ERα in breast cancers, still existed. Ascenzi et al. demonstrated that the context-specific capacity of tamoxifen binding with the extra-nuclear protein interactors accounted for the different transcriptional outcomes, where tamoxifen recruited transcriptional co-repressors in the breast and transcriptional co-activators in the endometrium (130). In ERα/GPER-positive estrogen-responsive breast cancer cell line MCF7, Zekas et al. identified that tamoxifen predominantly functioned as a GPER-selective agonist to rapidly transactivate EGFR and consequently inactivate GFP-fused Forkhead box O3 (FOXO3) in a GPER-mediated and ERα-independent manner on a transient time scale (131). Another study recently lent support to the carcinogenic role of tamoxifen, which was devoted to the [Ca2+] mobilization and overexpression of kinin B1 receptor, another G protein-coupled receptor (GPCR) demonstrated to facilitate proliferation and metastasis of breast cancer cells (132). In the context of GPER-positive cell lines irrespective of ERα, tamoxifen was endowed with an off-target effect that upregulated the aromatase expression by recruiting the c-fos/c-jun complex to responsive elements located in the promoter region of aromatase *via* GPER and sustained endocrine resistance (133).

The presented studies tend to recognize the non-classical ER-mediated mechanism of tamoxifen in TNBC, and tamoxifen should be repositioned to boost its adaption and application in the tumor environment of TNBC.

## Conclusions

Our work recapitulated the essential points by which the underappreciated endocrine network could be recharged and vitalized in an ERα-negative surrounding, and hormone analogs (including agonists and antagonists) play crucial roles in preventing carcinoma evolution, which proposed a fresh inspection into the hormone signal transduction and crosstalk with genetic codes. Complicated tumor microenvironments and intratumoral heterogeneity decreased the efficacy of traditional chemotherapy considerably in the receptor-deficient subtype, admittedly shedding light on the transitional and upgraded endocrine landscape of TNBC instead. Endocrine strategies could be interpreted with the medicine for maintenance and even strengthening during the interval of chemotherapy with chemotoxicity in recession, which awaits replication by future studies.

## Author Contributions

Conceptualization, CX. Writing—original draft preparation. CX. Writing—review and editing, OW. All authors have read and agreed to the published version of the manuscript.

## Conflict of Interest

The authors declare that the research was conducted in the absence of any commercial or financial relationships that could be construed as a potential conflict of interest.

## Publisher’s Note

All claims expressed in this article are solely those of the authors and do not necessarily represent those of their affiliated organizations, or those of the publisher, the editors and the reviewers. Any product that may be evaluated in this article, or claim that may be made by its manufacturer, is not guaranteed or endorsed by the publisher.

## Abbreviations

5αP, 5α-dihydroprogesterone; AR, Androgen receptor; AI, Aromatase inhibitors; BDNF, Brain-derived neurotrophic factor; BM, Brain metastasis; CSC, Cancer stem cell; CDKs, Cyclin-dependent kinases; DNMT, DNA methyltransferase; DNMTi, DNA methyltransferase inhibitor; ERα, Estrogen receptor α; ERRα, Estrogen-related receptor α; EREs, Estrogen response elements; GC, Glucocorticoids; GR, Glucocorticoid receptor; G6PD, Glucose-6-phosphate dehydrogenase; GnRHR, Gonadotropin-releasing hormone receptors; GnRHa, GnRH analogs; GPER, G-protein coupled estrogen receptor; GREs, Glucocorticoid response elements; Her2, Human epidermal growth factor receptor 2; IHC, Immunohistochemical staining; IL-6, Interleukin 6; LAR, Luminal-AR; mAR, membrane AR; mPRs, Membrane progesterone receptors; nPR, Nuclear progesterone receptor; OXPHOS, Oxidative phosphorylation; PGC-1α, Proliferator-activated receptor γ co-activator 1α; PGRMC1, Progesterone receptor membrane component 1; PR, Progesterone receptor; PRC, Polycomb repressor complexes; SERMs, Selective ER modulators; SERDS, Selective ER degraders; TAMs, Tumor-associated macrophages; TIME, Tumor immune microenvironment; TNBC, Triple-negative breast cancer; TrkB, Tropomyosin kinase receptor B; TP53INP1, Tumor protein 53-induced nuclear protein 1; VEGF, Vascular endothelial growth factor.
